# Immune effects of PI3K/Akt/HIF-1α-regulated glycolysis in polymorphonuclear neutrophils during sepsis

**DOI:** 10.1186/s13054-022-03893-6

**Published:** 2022-01-28

**Authors:** Tingting Pan, Shaoqiong Sun, Yang Chen, Rui Tian, Erzhen Chen, Ruoming Tan, Xiaoli Wang, Zhaojun Liu, Jialin Liu, Hongping Qu

**Affiliations:** 1 Department of Critical Care Medicine, Ruijin Hospital, Shanghai Jiao Tong University School of Medicine, 197 Ruijin Er Road, Shanghai, 200025 China; 2 Department of Emergency, Ruijin Hospital, Shanghai Jiao Tong University School of Medicine, 197 Ruijin Er Road, Shanghai, 200025 China

**Keywords:** Glycolysis, Immune, Metabolomics, Neutrophil, Sepsis

## Abstract

**Background:**

Effective removal of pathogenic bacteria is key to improving the prognosis of sepsis. Polymorphonuclear neutrophils (PMNs) are the most important components of innate cellular immunity and play vital roles in clearing pathogenic bacteria. However, the metabolic characteristics and immunomodulatory pathways of PMNs during sepsis have not been investigated. In the present study, we explored the immune metabolism characteristics of PMNs and the mechanism by which neutrophilic glycolysis is regulated during sepsis.

**Methods:**

Metabolomics analysis was performed on PMNs isolated from 14 septic patients, 26 patients with acute appendicitis, and 19 healthy volunteers. Transcriptome analysis was performed on the PMNs isolated from the healthy volunteers and the patients with sepsis to assess glycolysis and investigate its mechanism. Lipopolysaccharide (LPS) was used to stimulate the neutrophils isolated from the healthy volunteers at different time intervals to build an LPS-tolerant model. Chemotaxis, phagocytosis, lactate production, oxygen consumption rate (OCR), and extracellular acidification rate (ECAR) were evaluated.

**Results:**

Transcriptomics showed significant changes in glycolysis and the mTOR/HIF-1α signaling pathway during sepsis. Metabolomics revealed that the Warburg effect was significantly altered in the patients with sepsis. We discovered that glycolysis regulated PMNs’ chemotaxis and phagocytosis functions during sepsis. Lactate dehydrogenase A (LDHA) downregulation was a key factor in the inhibition of glycolysis in PMNs. This study confirmed that the PI3K/Akt-HIF-1α pathway was involved in the LDHA expression level and also influenced PMNs’ chemotaxis and phagocytosis functions.

**Conclusions:**

The inhibition of glycolysis contributed to neutrophil immunosuppression during sepsis and might be controlled by PI3K/Akt-HIF-1α pathway-mediated LDHA downregulation. Our study provides a scientific theoretical basis for the management and treatment of patients with sepsis and promotes to identify therapeutic target for the improvement of immune function in sepsis.

**Supplementary Information:**

The online version contains supplementary material available at 10.1186/s13054-022-03893-6.

## Introduction

Sepsis is defined as life-threatening organ dysfunction caused by dysregulated host response to infection [1]. It has become the most common cause of death in intensive care units (ICU) [2]. Pathogenic bacteria can not be promptly or effectively removed during sepsis [3], potentially resulting in multiple organ dysfunction aggravation and eventually, death in patients with sepsis [4]. Rapid and effective clearance of pathogenic bacteria are the key to improving the prognosis of sepsis.

Neutrophils are the most important components of innate cellular immunity [5]. When pathogenic bacteria invade the host, neutrophils arrive at the infection site to clear the infection [6]. In the early stages of sepsis, neutrophils are essential for clearing pathogenic bacteria and are the first line of defense [7, 8]. Neutrophils are multifaceted innate immunocytes that modulate the inflammatory response and initiate adaptive immune responses by releasing cytokines [9]. This coordinated response maintains immune homeostasis.

Neutrophilic dysfunction has been reported in patients with sepsis [10]. Previous studies consistently suggested that specific changes in neutrophil function occur in patients with sepsis; some of these are associated with poor clinical outcome [11]. A microarray analysis indicated the suppression of neutrophil immune and inflammatory function in patients with sepsis 24 h after admission [12]. Neutrophils may also have reduced antimicrobial function and impaired ability to inhibit adaptive immunity in vitro [13]. Hence, it is necessary to clarify the roles of neutrophils in dysregulated immune response that leads to deleterious outcomes in patients with sepsis.

Interest in metabolic reprogramming to regulate immune function has grown over the past decade. Recent studies on monocytes and lymphocytes have focused on metabolic adaptation [14, 15]. The roles of metabolic pathways in immune regulation have been clarified [16, 17]. Polymorphonuclear neutrophils (PMNs) have few mitochondria and may rely exclusively upon relatively inefficient glycolysis for energy metabolism, with glycolysis generating the majority of ATP required for neutrophil function [18, 19]. In the process of phagocytosis, ATP consumption rate is very high [20], and in sepsis, systemic ATP inhibits the activation and chemotaxis of neutrophils by interfering with the endogenous purinergic signaling mechanism [21]. However, the metabolic characteristics and immunomodulatory pathways of neutrophils during sepsis have not yet been investigated.

As the present study focuses on sepsis-related neutrophil dysfunction, we compared the metabolic properties of the neutrophils in patients with and without sepsis. We examined the metabolic characteristics and mechanisms of neutrophils in sepsis. We also explored the possible relationship between immune function and metabolism in neutrophils during sepsis.

## Materials and methods

### Reagents and antibodies

PolymorphPrep™ was obtained from Axis-shield AS (Oslo, Norway). HK2, HK3, PKM2, LHDA, Akt, p-Akt, PI3k, p-PI3k, and HIF-1α antibodies were purchased from Cell Signaling Technology (Danvers, MA, USA). Lactate estimation kits were obtained from Jiancheng Chemical (Nanjing, China). RPMI 1640 medium, fetal bovine serum (FBS), Quant-iT™ PicoGreen dsDNA assay kits, and Sytox Green were acquired from Invitrogen (Carlsbad, CA, USA). Insulin, 2-DG, LY294002, BAY-85, BAY-87 and other chemicals were purchased from Sigma Aldrich Corp. (St. Louis, MO, USA) unless otherwise specified. Detailed information about these reagents and antibodies is shown in Additional file 1: Table S1.

### Study participants

All participants provided written informed consent and the study was performed in accordance with the principles of the Declaration of Helsinki. Fourteen patients admitted to the emergency department or ICU of Ruijin Hospital between July 2018 and July 2019 were enrolled in this study within 24 h after the diagnosis of sepsis. Sepsis 3.0 criteria were used to define sepsis [1]. Patients were assigned to the sepsis group if they had life-threatening organ dysfunction indicated by an increase of at least two points in the Sequential Organ Failure Assessment (SOFA) score after infection. The study exclusion criteria were HIV infection, autoimmune disease, hematological neoplasms, and viral hepatitis. Blood samples were collected on the first day of diagnosis of sepsis. In order to analyze the differences between the non-septic infection group and the septic group, we used two control groups: (1) a healthy control group comprised of healthy volunteers, (2) a non-septic infection group (infection group without organ dysfunction) comprised of patients with acute appendicitis. Both control groups were matched by age and sex. The detailed characteristics of the participants are listed in Additional file 2: Table S2.

### Human neutrophil isolation

Venous blood was drawn from patients and healthy adult volunteers and immediately transferred to tubes containing EDTA. Neutrophils were isolated from whole blood with PolymorphPrep™ (Axis-shield AG, Oslo, Norway). Five milliliters PolymorphPrep™ was placed in a 15-mL round-bottom tube and 5 mL whole blood was layered onto the PolymorphPrep™. This preparation was then centrifuged at 500 × *g* and 20 °C for 30 min to separate the blood into its components. The granulocyte layer was harvested, resuspended in phosphate-buffered saline (PBS), and washed by centrifugation at 350 × *g* for 10 min. The red blood cells were lysed. The neutrophils were washed, counted with a hemocytometer, and centrifuged at 350 × *g* for 10 min. The neutrophils were then diluted to the required concentration in RPMI 1640 medium (Gibco Ltd., Grand Island, NY, USA) supplemented with 10% fetal bovine serum (FBS) or cryopreserved in liquid nitrogen until subsequent analysis. Differential counts showed that all preparations consisted of > 97% neutrophils and > 95% of them were viable according to the Trypan blue dye exclusion assay.

### Transcriptomics analysis

RNA was collected with the RNeasy Micro kit (Qiagen, Hilden, Germany) from the PMNs of healthy controls and patients with sepsis. Total RNA quality was assessed by spectrophotometry (NanoDrop; Thermo Fisher Scientific Inc., Waltham, MA, USA) and an Agilent 4200 Bioanalyzer (Agilent Technologies, Palo Alto, CA, USA). Intact mRNA was isolated with a Dynabead mRNA purification kit for total RNA (Thermo Fisher Scientific, Waltham, MA, USA) according to the manufacturer’s protocol. Amplified cDNA was prepared with a NEB Next8.1 Poly(A) mRNA magnetic isolation module (New England Biolabs, Ipswich, MA, USA) according to the manufacturer’s protocol. Sequencing libraries were generated with the Nextera XT library preparation kit and multiplexing primers (Illumina, San Diego, CA, USA) according to the manufacturer’s protocols. Library fragment size distributions were assessed with the Bioanalyzer 4200 and a DNA high-sensitivity chip (Agilent Technologies, Santa Clara, CA, USA). Library sequence quality was assessed by sequencing single-end, 50-bp reads on the Illumina MiSeq platform (Illumina, San Diego, CA, USA). Libraries were pooled for high-throughput sequencing on the Illumina NovaSeq 6000 (Illumina, San Diego, CA, USA) using equal numbers of uniquely mapped protein-coding reads. RNA sequencing was performed on NovaSeq 6000 machines (Illumina, San Diego, CA, USA). The raw sequencing results were then de-multiplexed, trimmed of adapter sequences, and aligned to the reference genome using STAR v. 49. DESeq2 was used for normalization by size factor (reads per sample) and library complexity. The Wald test was used to determine the significance of differential expression [22].

### Metabolomics analysis

We performed the metabolomics analysis with a Q300 Kit (Metabo-Profile, Shanghai, China). Harvested cell samples were stored in an Eppendorf Safelock microcentrifuge tube (Eppendorf, Hamburg, Germany) and mixed with ten pre-chilled zirconium oxide beads and 20 µL deionized water. Samples were homogenized for 3 min and 150 µL methanol containing the internal standard was added to extract the metabolites. The samples were homogenized for another 3 min and centrifuged at 18,000 × *g* for 20 min. The supernatants were transferred to 96-well plates. The following procedures were performed on a Biomek 4000 workstation (Biomek 4000; Beckman Coulter Inc., Brea, CA, USA). Twenty microliters freshly prepared derivative reagent was added to each well. The plate was sealed and derivatization was conducted at 30 °C for 60 min. After derivatization, the samples were evaporated for 2 h and 330 µL ice-cold 50% (v/v) methanol was added to each well to reconstitute the samples. The plate was stored at − 20 °C for 20 min and the samples were centrifuged at 4000 × *g* and 4 °C for 30 min. Then, 135 µL supernatant was transferred to a new 96-well plate. Each well contained 10 µL internal standards. Serial dilutions of derivatized stock standards were added to the wells on the left side and the plate was sealed for liquid chromatography-mass spectrometry (LC–MS) analysis. All internal standards were obtained from Sigma-Aldrich Corp. (St. Louis, MO, USA), Steraloids Inc. (Newport, RI, USA), and TRC Chemicals (Toronto, ON, Canada). All standards were accurately weighed and prepared in water, methanol, aqueous sodium hydroxide, or aqueous hydrochloric acid to obtain 5.0 mg/mL stock solutions. Appropriate amounts of each stock solution were mixed to prepare stock calibration solutions. An ultraperformance liquid chromatography coupled to tandem mass spectrometry (UPLC-MS/MS) system (ACQUITY UPLC-Xevo TQ-S; Waters Corp., Milford, MA, USA) was used by Metabo-Profile Biotechnology (Shanghai) Co. Ltd. to quantitate all metabolites targeted in the present study.

Raw data files generated by UPLC-MS/MS were processed on the iMAP platform (v. 1.0; Metabo-Profile, Shanghai, China). Principal component analysis (PCA) and orthogonal partial least squares discriminant analysis (OPLS-DA) were also conducted. Variable importance in projection (VIP) was obtained based on the OPLS-DA model. Metabolites with VIP > 1 and *P* < 0.05 were considered as significantly differentially expressed metabolites (DEMs). The Z-score indicates the number of standard deviations by which an observation is above or below the control group mean, the V-plot integrates the fold change, and the P-value indicates significantly different metabolites.

### In vitro cell model

For the lipopolysaccharide (LPS)-activated and LPS-tolerant models, primary neutrophils were cultured in RPMI 1640 medium supplemented with 10% FBS. The cells were treated with two concentrations of LPS (100 ng/mL and 1 ug/mL). Chemotaxis, phagocytosis, and lactate production were evaluated at 0 h, 1 h, 2 h, 4 h, 6 h, 8 h, and 12 h to determine the phase points of neutrophil activation and inhibition.

### Lactate quantification assay

Lactate accumulation in the culture was assessed with a commercially obtained lactate estimation kit (Nanjing Jiancheng, China) according to the manufacturer’s instructions.

### Neutrophil chemotaxis assay

Neutrophil chemotaxis was measured in a 96-well chemotaxis chamber (Neuroprobe Inc., Gaithersburg, MD, USA) using the method of Frevert et al. [23] with modifications. Wells were filled with fMLP (50 nM), RPMI 1640 medium, or neutrophils (5 × 10^4^) resuspended in RPMI 1640 medium. A filter membrane was positioned over the loaded wells and 25 μL neutrophils (2 × 10^6^/mL) was placed directly onto 3.0-μm filter sites. The chamber was incubated under 5% CO_2_ at 37 °C for 1 h. Unmigrated neutrophils were removed from the upper surface of the filter by wiping and washing with 25-μL aliquots of RPMI 1640 medium. Neutrophils that migrated to the underside of the filter and into the lower wells were counted with a hemocytometer. To dislodge any migrated cells adherent to the underside of the filter membrane, the plate and attached filter were centrifuged at 350 × *g* for 10 min. The filter was removed and the neutrophils in the wells of the chemotaxis plate were resuspended and counted with a hemocytometer.

### Neutrophil phagocytosis assay

For Neutrophil Phagocytosis Assay, a flow cytometric technique was used to detect phagocytosis, as reported previously [24, 25]. Aliquots of 100 μL of cells at a concentration of 1 × 10^4^/μL were incubated with 10 μL of FluoSpheres Fluorescent Microsphere (1 × 10^10^ microspheres/mL Invitrogen F13081) for 40 min at 37 °C. (In this procedure, neutrophils ingest the microsphere particles through phagocytosis.) Next, the cells were washed five times with PBS (to remove the free particles) and then suspended in 1 ml of PBS. The cell suspensions were analyzed using a flow cytometer (Becton Dickinson, NJ, USA).

### Annexin V-FITC/PI FACS (fluorescence-activated cell sorting) apoptosis assay

Cells were trypsinized with an Annexin V-FITC/PI apoptosis detection kit (Solarbio Life Sciences, Beijing, China) according to the manufacturer’s instructions and then washed twice with PBS. The cells were resuspended in a mixture of 200 μL binding buffer, 10 μL Annexin V-FITC, and 10 μL PI, gently mixed, and incubated in the dark at 37 °C for 15 min. Binding buffer (300 μL) was added to each tube and the samples were analyzed by flow cytometry (Becton Dickinson, Franklin Lakes, NJ, USA) within 1 h.

### Real-time cell metabolism assay

The XFp Extracellular Flux Analyzer (Seahorse Bioscience, North Billerica, MA, USA) was used for real-time analysis of the extracellular acidification rate (ECAR) and the oxygen consumption rate (OCR). Briefly, human neutrophils (5 × 10^4^/well) were resuspended in sterile XF base media supplemented with 10 mM *D*-glucose (pH 7.4), plated on XFp cell culture plates pre-coated with 0.001% poly-*L*-lysine, and allowed to settle at 37 °C for 30 min. The manufacturer’s instructions were followed to obtain real-time ECAR and OCR measurements. OCR was measured under the following conditions: (1) basal; (2) 1 μM oligomycin; (3) 0.3 μM FCCP; and (4) 0.5 μM rotenone + 0.5 μM antimycin. To quantify ECAR, the glycolysis inhibitor 2-DG was injected to stop glycolytic acidification. OCR and ECAR were normalized to the total protein.

### Western blot

Proteins from the cultured cells were obtained using cell lysis buffer (50 mM Tris (pH 8.0), 150 mM NaCl, 1% (w/v) NP-40, and 0.1% (w/v) SDS). Protein concentrations were measured with a bicinchoninic acid (BCA) assay kit (Thermo Fisher Scientific, Waltham, MA, USA). The protein extracts were denatured at 100 °C for 5 min and separated by 10% sodium dodecyl sulfate–polyacrylamide gel electrophoresis (SDS-PAGE) at 80 V for ~ 1.5 h. Proteins were blotted onto an Immobilon-P™ polyvinylidene fluoride (PVDF) membrane (Merck Millipore Ltd., Dublin, Ireland) at 100 V for 2 h. The membranes were blocked with 5% (v/v) bovine serum albumin (BSA) in Tris-buffered saline with 0.1% (w/v) Tween-20 (TBST) at room temperature for 30 min. The membranes were then incubated overnight at 4 °C with primary antibodies to various candidate proteins (1:1,000). The membranes were washed thrice with TBST. Specific horseradish peroxidase (HRP)-conjugated secondary antibody was added at 1:5000 and detected by enhanced chemiluminescence (ECL). The optical density was then measured by BioRad ChemiDox (BioRad Laboratories, Hercules, CA, USA).

### Statistical analyses

Data are presented as means ± standard deviation (SD) for at least three independent experiments. Statistical analyses were performed using GraphPad Instat v. 3.01 (GraphPad Software, San Diego, CA, USA). Means of two groups were compared using Student’s *t*-test. One-way ANOVA with Brown-Forsythe and Welch ANOVA tests were performed for multiple comparisons. *P* < 0.05 was considered statistically significant. *P < 0.05, **P < 0.01, and ***P < 0.001.

## Results

### PMN transcriptomics indicated significant changes in glycolysis and the mTOR/HIF-1α signaling pathway during sepsis

**Fig. 1:**
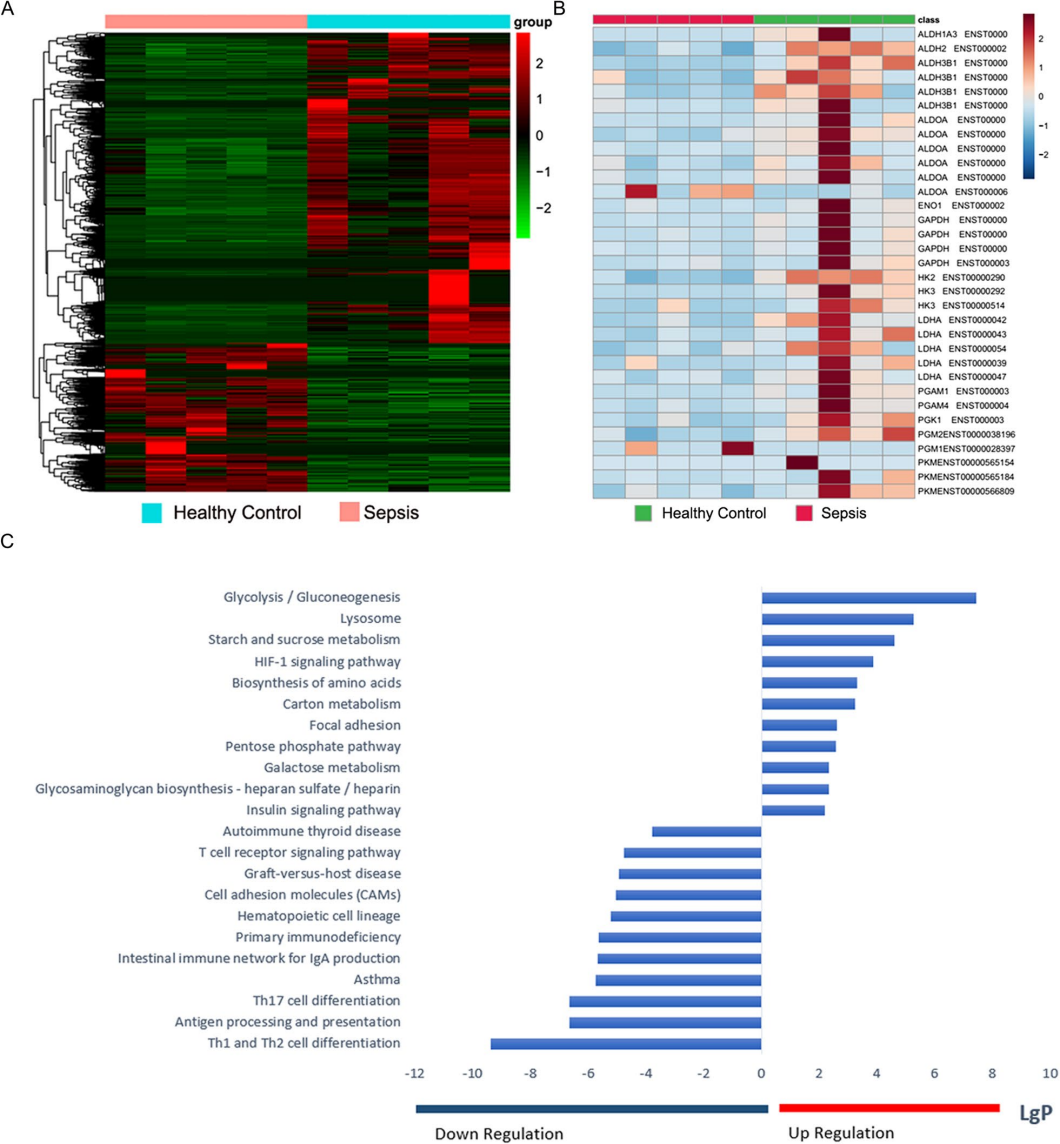
PMN transcriptomics revealed significant changes in glycolysis and mTOR/HIF-1α signaling pathway. **a** Heat map of DEGs identified by RNA-seq between patients with sepsis and healthy controls (*P* < 0.05). **b** Differential expression levels of glycolysis-associated genes in patients with sepsis versus those in healthy controls. **c** Histogram of KEGG enrichment analysis showing 22 significantly enriched pathways in patients with sepsis. KEGG ontology assignments were used to classify functional annotations of identified genes to elucidate their biological functions

An objective of the present study was to explore the immune function and metabolic characteristics of PMNs in sepsis. We examined the transcriptomes of PMNs isolated from healthy volunteers and patients with sepsis to assess glycolysis and its mechanism in PMNs. RNA-Seq returned ~ 39 M raw reads per sample. We reviewed the raw data of the RNA-seq and found that the integrity of the RNA met the quality control standards. The representative diagram has been shown as Additional file 3: Fig. S1. After quality filtration, ~ 95.6% of them were mapped as clean reads. The threshold of significance was set to a false discovery rate (FDR) < 0.05 and 677 differentially expressed genes (DEGs) were retained (Additional file 4: Table S3) as shown in a heat map (Fig. 1a). Gene ontology (GO) term and Kyoto Encyclopedia of Genes and Genomes (KEGG) pathway assignments were used to identify the gene functions affected by sepsis. KEGG pathway assignments were determined by mapping with the KEGG database and identified 224 significantly (*P* < 0.05) enriched genes spanning 88 pathways (Additional file 5: Table S4). Genes encoding the proteins involved in glycolysis and the hypoxia-inducible factor (HIF)-1α pathway were significantly upregulated in the PMNs from patients with sepsis relative to those of healthy volunteers (Fig. 1c). The glycolysis-encoding genes *HK2* (hexokinase-2), *HK3* (hexokinase-3), *LDHA*, and *PKM* (pyruvate kinase M) in the PMNs of patients with sepsis were significantly altered compared to those in the PMNs of healthy volunteers (Fig. 1b).

### PMN spectra and metabolic profiles

We further explored the metabolic characteristics of the PMNs from patients with or without sepsis. The study included three groups of patients: (1) septic; (2) non-septic, including patients with acute appendicitis and no organ function impairment; and (3) healthy volunteers. Patients with sepsis and acute appendicitis were diagnosed within 24 h. Clinical characteristics of the groups are listed in Additional file 2: Table S2.

**Fig. 2:**
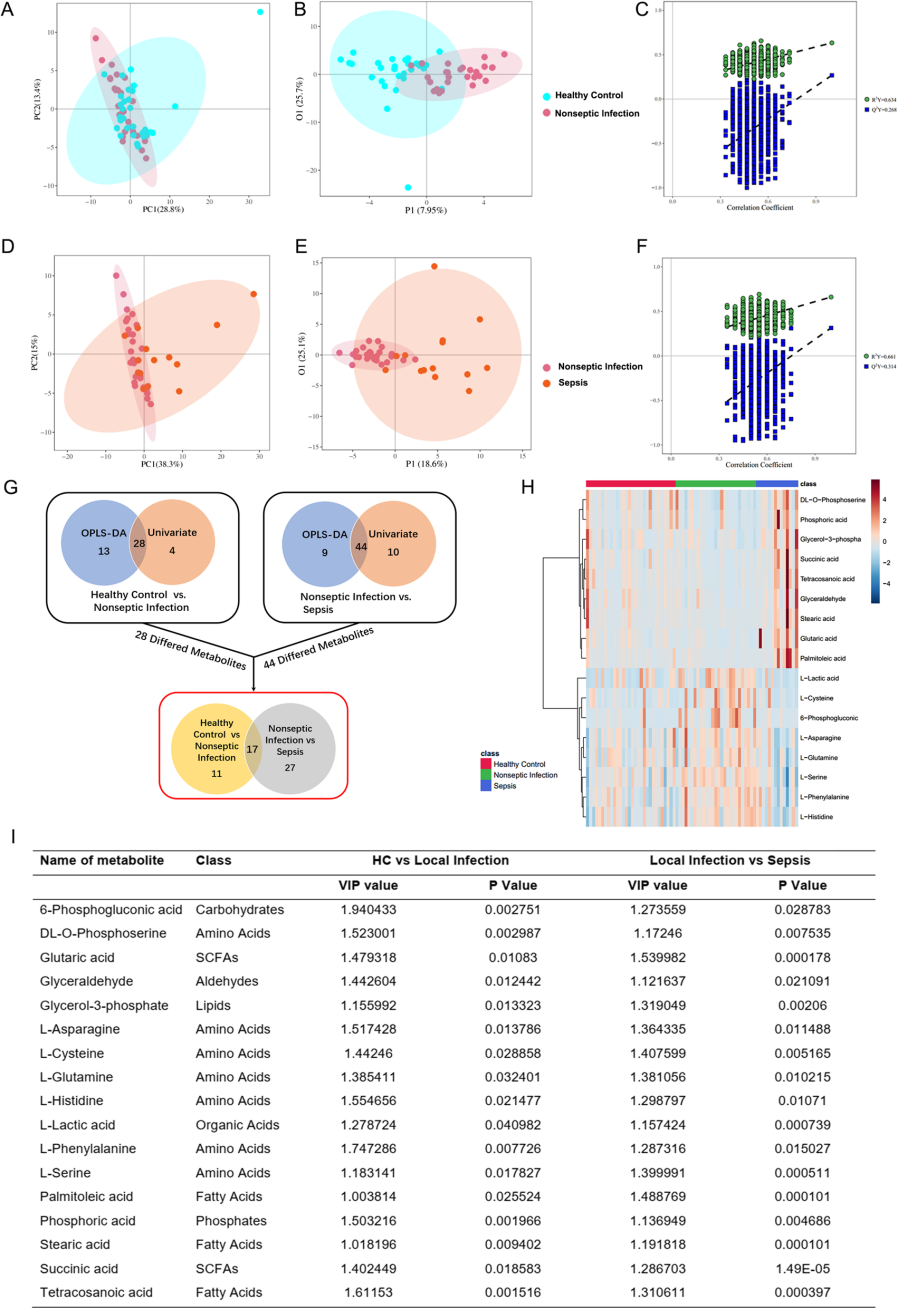
Multivariate metabolite data analysis. **a**, **b** Principal component analysis (PCA) scores plots (left panels) and orthogonal partial least square-discriminant analysis (OPLS-DA) scores plots (right panels) of patients with non-septic infection versus those of healthy controls. Shaded areas are 95% confidence interval (CI) regions of each group. **c** Permutation test for OPLS-DA model of patients with non-septic infection versus that of healthy controls. **d**, **e** PCA and OPLS-DA score plots of patients with non-septic infection versus those of patients with sepsis. Shaded areas are 95% confidence interval (CI) regions of each group. **f** Permutation test for OPLS-DA model of patients with non-septic infection versus that of patients with sepsis. **g** Schematic diagram of data analysis procedures. **h** Heatmap showing abundance of 17 metabolites based on VIP scores of patients with sepsis, patients with non-septic infection, and healthy controls. **i** Differentiated metabolites among patients with sepsis, patients with non-septic infection, and healthy controls

PMNs from all three groups were analyzed by untargeted high-resolution metabolomics and > 1200 metabolite properties were detected. Missing values were filtered, and 110 metabolite properties were retained for statistical analysis. Differential metabolomic profiles of non-septic patients versus healthy controls and septic patients versus non-septic patients were obtained via principal component analysis (PCA) and orthogonal partial least square-discriminant analysis (OPLS-DA). The score plots displayed significant separation between non-septic patients (*R*^2^ = 0.634; *Q*^2^ = 0.268) and healthy controls as well as between patients without sepsis and those with sepsis (*R*^2^ = 0.661; *Q*^2^ = 0.314) (Fig. 2a–f). We screened differential metabolites by selecting those with *P* < 0.05 (Student’s t-test) and VIP > 1.0 (OPLS-DA model). A schematic illustration of the data analysis method is provided. There were twenty-eight DEMs in the non-septic group versus the healthy control group, and forty-four DEMs in the non-septic group versus the septic group. Seventeen DEMs overlapping in these three groups were presented in Fig. 2g and i. The heatmap showed the relative abundance of all 17 common differential metabolites in all individuals based on the VIP scores (Fig. 2h). We found that the lactic acid levels in the neutrophils were significantly higher in the non-septic patients than that in the healthy controls (*P* = 0.04). In contrast, the lactic acid levels in the neutrophils were significantly lower in the septic patients compared to that in the non-septic patients (*P* = 0.0007).

## Warburg effect was significantly altered in the patients with sepsis

**Fig. 3:**
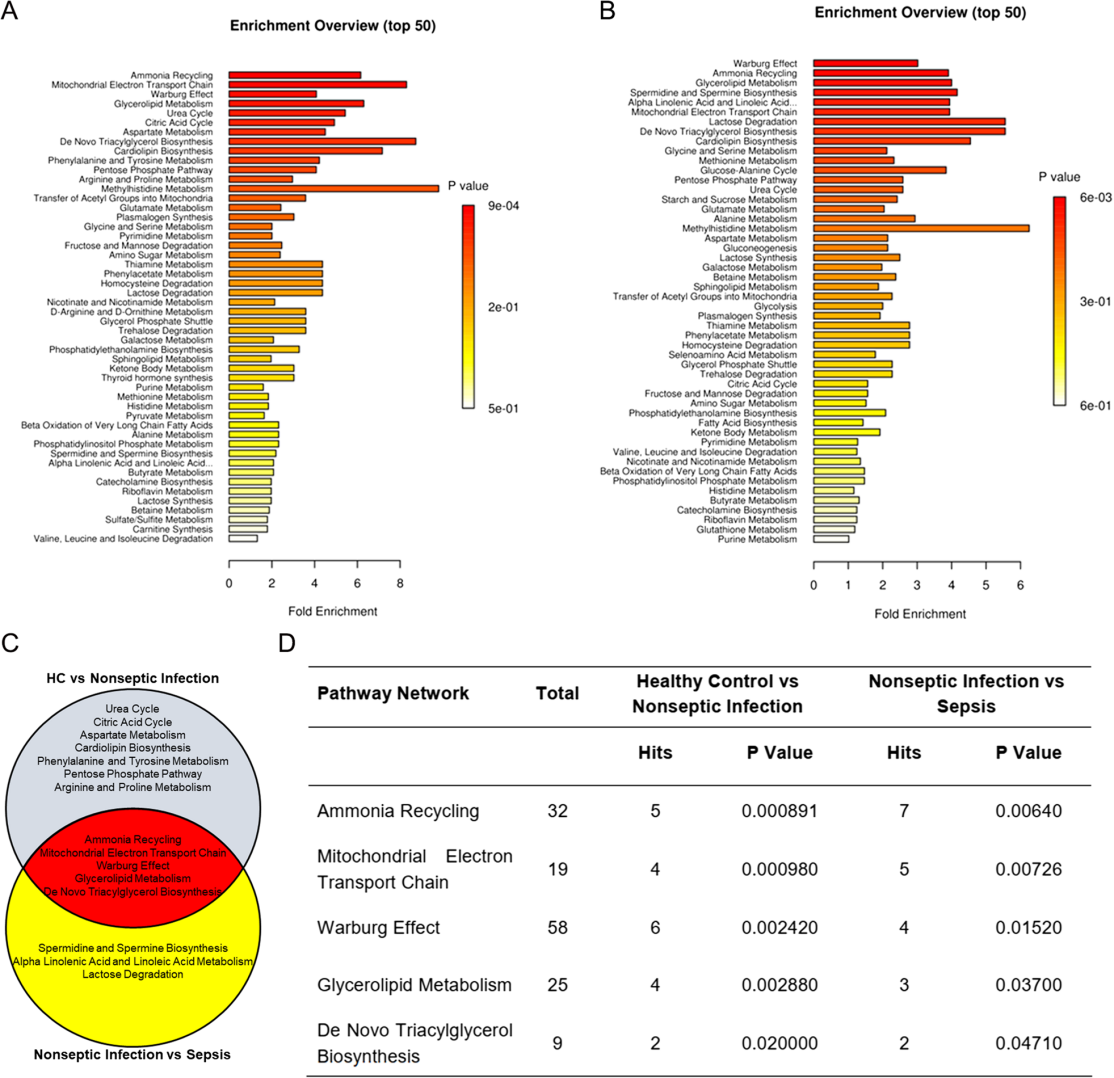
Pathway analysis showing that the Warburg Effect pathway was significantly changed in patients with sepsis. **a** Pathway analysis revealed altered pathways in patients with non-septic infection versus those in healthy controls. **b** Pathway analysis revealed altered pathways in patients with non-septic infection versus those in patients with sepsis. **c**, **d** Pathways altered in patients with non-septic infection versus those in healthy controls (white circle) and in patients with non-septic infection versus those patients with sepsis (yellow circle). Pathways in cross area (red circle) were identified in both groups

We compared the pathways between the septic patients and the healthy controls (Fig. 3a). We also compared the pathways between the non-septic patients with the patients with sepsis (Fig. 3b). Figure 3c showed the enriched pathways that overlapped in the healthy control group, the non-septic group, and the septic group. The most significantly enriched pathways included the Warburg Effect, Mitochondrial Electron Transport Chain, Ammonia Recycling, Glycerolipid Metabolism, and De Novo Triacylglycerol Biosynthesis (Fig. 3d).

### The inhibition of neutrophil glycolysis was accompanied with immune dysfunction in the LPS-tolerant model in vitro

**Fig. 4:**
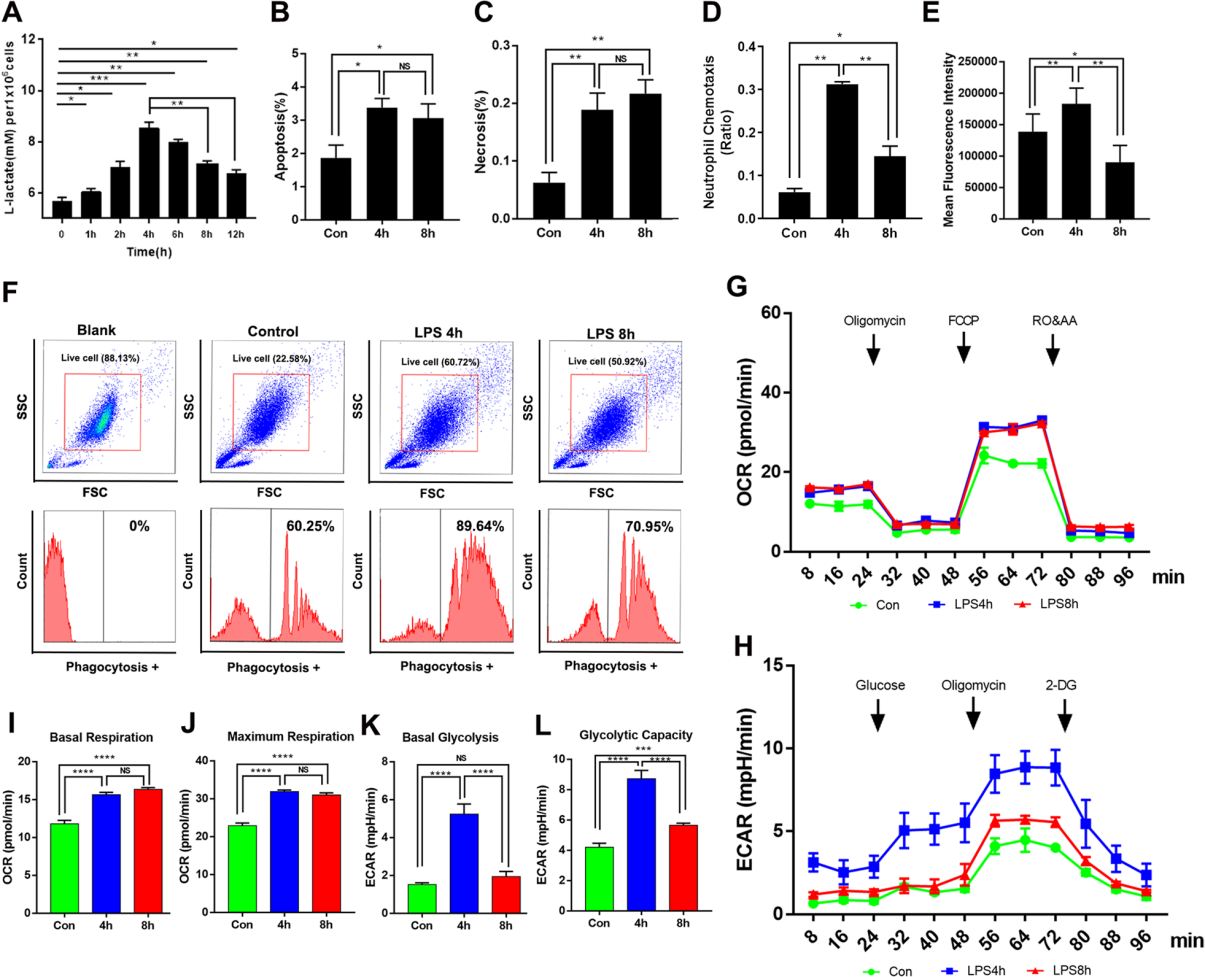
Glycolysis in neutrophils is inhibited and accompanied by immune dysfunction in LPS-tolerant model. To build the LPS-tolerant model, PMNs were treated with LPS (100 ng/mL) for 4 h or 8 h. **a** L-lactate concentrations in untreated PMNs and in those stimulated with LPS for 4 h and 8 h. **b** Apoptosis and **c** necrosis in untreated PMNs and in those stimulated with LPS for 4 h and 8 h. **d** Chemotaxis and **e** Phagocytosis in culture media of untreated PMNs and those stimulated with LPS for 4 h and 8 h. **f** Representative flow cytometry images of neutrophils stained with FITC and showing MFI in untreated PMNs and those stimulated with LPS for 4 h and 8 h. Neutrophils were seeded in Seahorse XFp analyzer culture plates (Seahorse Bioscience, North Billerica, MA, USA) and real-time OCR (**g**, **i**, **j**) and ECAR (**h**, **k**, **l**) were measured. ECAR reflected lactate production in untreated PMNs and those stimulated with LPS for 4 h and 8 h. One-way ANOVA with Brown-Forsythe and Welch ANOVA tests were performed. Error bars represent SD. Data are means ± SD of at least three independent experiments. *P < 0.05, **P < 0.01, ***P < 0.001

Using metabolomics analysis, we observed that the Warburg effect was significantly altered in patients with sepsis compared with that in non-septic patients. Chemotaxis and phagocytosis were used to evaluate the immune function of neutrophils from healthy controls, non-septic patients, and septic patients. The neutrophils from the non-septic patients showed significantly higher levels of chemotaxis and phagocytosis compared to healthy controls. However, when compared to the non-septic patients, the levels of chemotaxis and phagocytosis in the neutrophils of septic patients were significantly lower (Additional file 6: Fig. S2). To explore the mechanism of immunometabolism in PMNs during sepsis, we used LPS to stimulate neutrophils for different time periods and established the LPS-tolerant model. It has been widely recognized that continuous LPS stimulation induces tolerance of defensive or allergic responses such as fever, shock [26], and inflammatory cytokine production in the host. Since LPS tolerance was first described, many studies have reported on the hyporesponsiveness to LPS in vitro based on the attenuation of proinflammatory cytokine production [27]. We used two concentrations of LPS (100 ng/mL and 1ug/mL) to continuously stimulate primary neutrophils over time, the relevant chart has been shown in Fig. 4a and Additional file 7: Fig. S3. As shown in Additional file 6: Fig. S2, the mRNA levels of TNF⍺, IL-6, CX3CR1, and CCL2 were significantly lower in neutrophils stimulated with LPS for 8 h compared to that in neutrophils stimulated with LPS for 4 h. In addition, the levels of chemotaxis and phagocytosis were both significantly lower in the 8 h treated neutrophils than that in the 4 h treated cells (Fig. 4d–f). Hence, 4 h and 8 h LPS-stimulated neutrophils were taken as the LPS-activated and LPS-tolerant cell models, respectively. Although Neutrophils have short lifespans in peripheral blood, certain studies have shown that stimulated neutrophils may live relatively longer than unstimulated ones [28, 29]. In addition, no significant differences were shown between LPS-tolerant and LPS-activated neutrophils in terms of apoptosis or necrosis rate (Fig. 4b, c).

The lactic acid level peaked at 4 h after LPS stimulation and decreased thereafter (Fig. 4a). To understand the metabolic changes that occur in neutrophil activation, we stimulated neutrophils with LPS for 4 h or 8 h and evaluated real-time changes in oxygen consumption rate (OCR) and extracellular acidification rate (ECAR) in a Seahorse system (Seahorse Bioscience, North Billerica, MA, USA). ECAR is an index of lactate production. After 4 h LPS stimulation, both OCR and ECAR had dramatically increased in the neutrophils (Fig. 4g–l). After 8 h LPS stimulation, all LPS-tolerant neutrophils exhibited similar OCR whereas their ECAR had significantly decreased (Fig. 4g–l). Whereas the PMNs switched towards aerobic glycolysis in response to activation, glycolysis was inhibited in the LPS-tolerant neutrophils.

### Glycolysis regulates PMN chemotaxis and phagocytosis function during sepsis

**Fig. 5:**
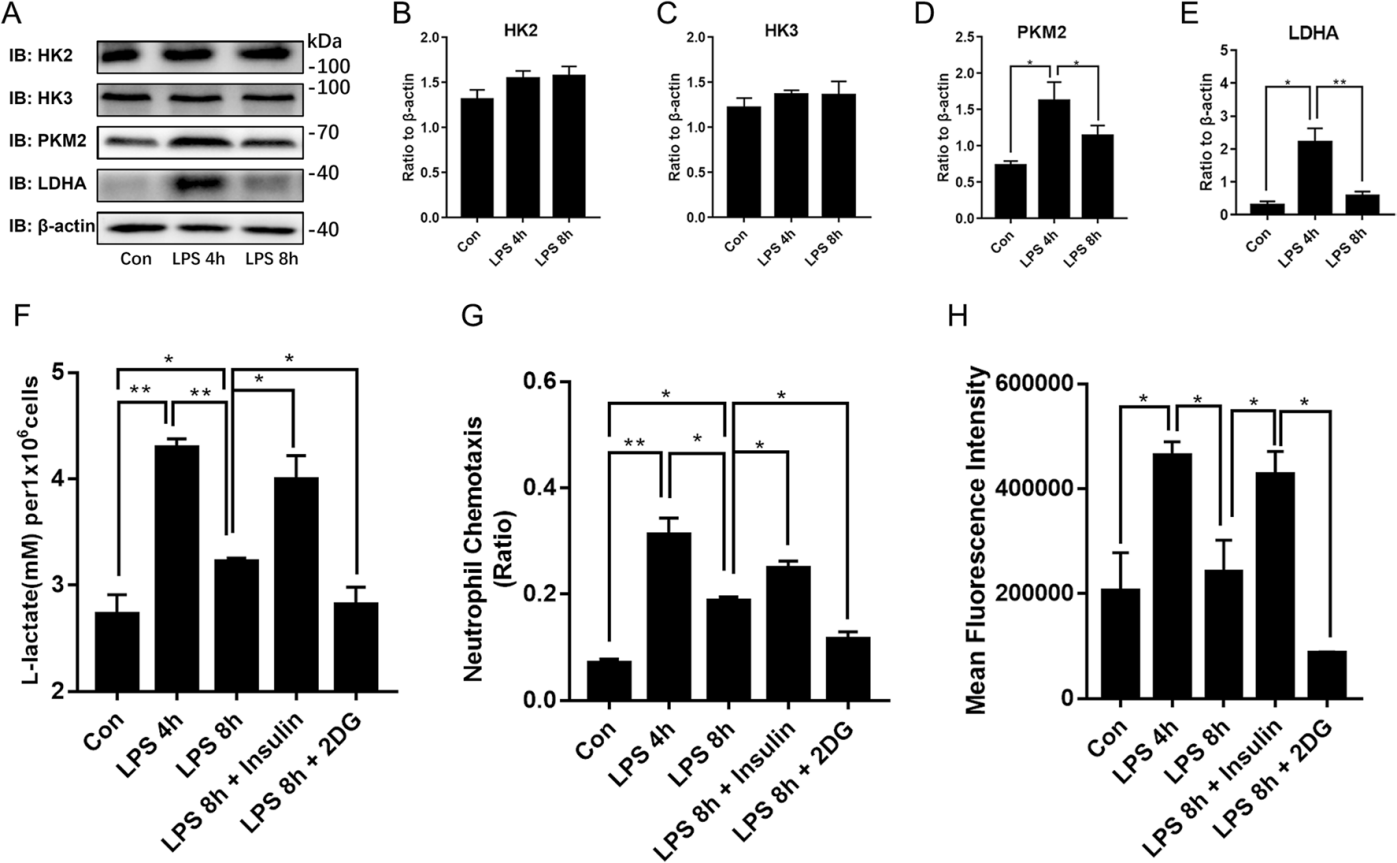
Glycolysis regulates PMNs’ chemotaxis and phagocytosis functions during sepsis. PMNs were treated with LPS (100 ng/mL) for 4 h or 8 h to build LPS-activated and LPS-tolerant models. **a–e** Hexokinase2 (HK2), hexokinase3 (HK3), M2 isoform of pyruvate kinase (PKM2), and lactate dehydrogenase A (LDHA) expression levels were measured by western blot. Neutrophils were pretreated with 100 μM insulin or 1 mM 2-DG for 30 min and then incubated with LPS for 4 h or 8 h. **f** *L*-lactate concentrations in untreated PMNs and those stimulated with LPS for 4 h and between PMNs pretreated with 100 μM insulin or 1 mM 2-DG for 30 min before incubation and those subjected to LPS for 8 h. **g** PMN chemotaxis. **h** PMN phagocytosis. Images are representative of three independent experiments. One-way ANOVA with Brown-Forsythe and Welch ANOVA tests were performed. Error bars represent SD. Data are means ± SD of at least three independent experiments. *P < 0.05, **P < 0.01

HK2, HK3, LDHA, and PKM were screened by transcriptomics and verified by western blot to clarify their involvement in the regulation of glycolysis in neutrophils. After 4 h LPS stimulation, LDHA and PKM were upregulated in the PMNs compared to the control. After 8 h LPS stimulation, LDHA and PKM were downregulated compared with their expression levels in response to 4 h LPS stimulation (Fig. 5a, d, f). However, there were no significant differences between HK2 and HK3 in terms of their relative expression levels in response to 4 h or 8 h LPS stimulation (Fig. 5a–c).

We used the glycolysis inhibitor 2-DG (2-deoxyglucose) to establish the roles of glycolysis in PMNs’ chemotaxis and phagocytosis functions during sepsis. In LPS-tolerant neutrophils, 2-DG reduced lactate production (Fig. 5f), neutrophil chemotaxis (Fig. 5g), and phagocytosis (Fig. 5h) compared with LPS-tolerant neutrophils without 2-DG treatment. In contrast, insulin increased the lactate production, chemotaxis, and phagocytosis of neutrophils (Fig. 5f–h).

To clarify the role of LDHA in phagocytosis, we used LDHA inhibitors (FX-11) to explore the role of LDHA in primary neutrophils. FX-11 was found to be a potent, competitive inhibitor of the enzyme’s NADH binding pocket [30]. As shown in the Additional file 8: Fig. S4, phagocytosis of neutrophils was significantly inhibited by FX-11 pretreatment. In addition, we developed LPS-activated/tolerant model with the neutrophil-like secondary cell line (HL-60 cells). Different from the primary neutrophils, phagocytosis and lactate level were significantly lower in the 6 h LPS-treated group than in the 2 h LPS-treated group. Therefore, 2 h and 6 h LPS-treated neutrophils were used as the LPS-activated and LPS-tolerant cell models. We found that knock down of LDHA significantly reduced the phagocytosis level of neutrophils upon 2 h LPS stimulation (Additional file 8: Fig. S4). These results indicated that LDHA plays an important role in regulating the phagocytosis of neutrophils.

### LPS stimulation decreased PMN glycolysis level via PI3K/Akt/HIF-1α pathway

**Fig. 6:**
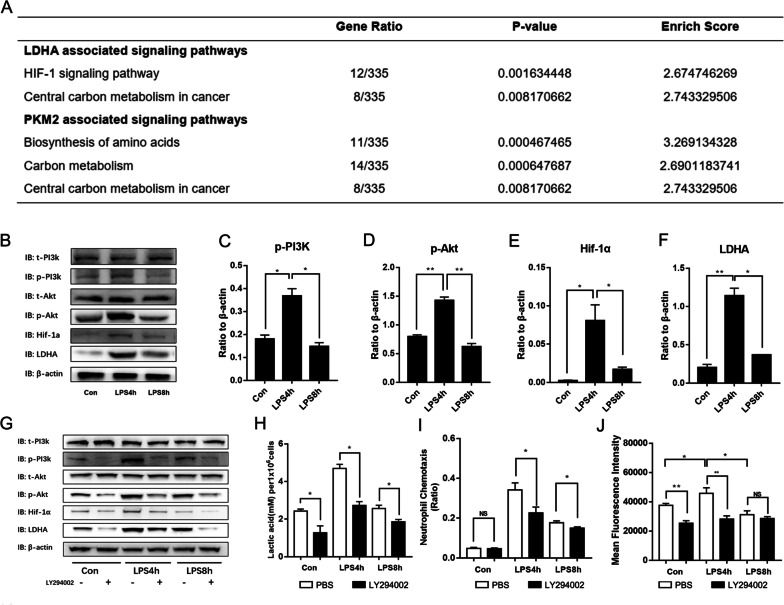
PI3K/Akt/HIF-1α pathway is responsible for the decrease in PMN glycolysis during sepsis. **a** Schematic representation of enzymes transcriptomically associated with the PI3K/Akt/HIF-1α signaling pathway. **b** Western blot for untreated PMNs and those stimulated with LPS for 4 h and 8 h. Endogenous phospho-PI3K, total PI3K, phospho-Akt, total Αkt, ΗΙF-1α, LDHA, and actin. **c**–**f** Representative bar chart of p-PI3K/t-PI3K, p-Αkt/t-Αkt, ΗΙF-1α/β-actin, and LDHA/β-actin ratios. To examine the effects of PI3K/Akt pathway on PMN glycolysis during sepsis, PMNs were stimulated with LPS for 4 h or 8 h in the presence of LY294002 (LY; 10 mM). Cell lysates were prepared at the indicated time points. **g** Western blot for endogenous p-PI3K, t-PI3K, p-Akt, t-Αkt, ΗΙF-1α, LDHA, and actin. **h** *L*-lactate concentrations in PMNs. **i** PMN chemotaxis. **j** PMN phagocytosis. Images are representative of three independent experiments. One-way ANOVA with Brown-Forsythe and Welch ANOVA tests were performed. Error bars represent SD. Data are means ± SD of at least three independent experiments. *P < 0.05, **P < 0.01

PMN transcriptomics identified HIF-1α as the LDHA-associated signaling pathway in neutrophils during sepsis (Fig. 6a). To validate this, we found that 4 h LPS stimulation dramatically upregulated the levels of phosphorylated PI3K and Akt as well as the protein levels of HIF-1α and LDHA in the neutrophils, which were significantly higher than that in the LPS-tolerant neutrophils (Fig. 6b–f).

To investigate the effects of PI3K/Akt pathway on PMN glycolysis during sepsis, PMNs were stimulated with LPS for 4 h or 8 h in the presence of the PI3K/Akt pathway inhibitor LY294002. LY294002 pretreatment markedly reduced the phosphorylation levels of PI3K and Akt as well as the protein levels of HIF-1α and LDHA (Fig. 6g). In addition, lactate production, neutrophil chemotaxis, and phagocytosis also substantially inhibited by LY294002 (Fig. 6h–j). These data suggested that PI3K/Akt inhibition contributes to the decrease in PMN glycolysis during sepsis.

**Fig. 7:**
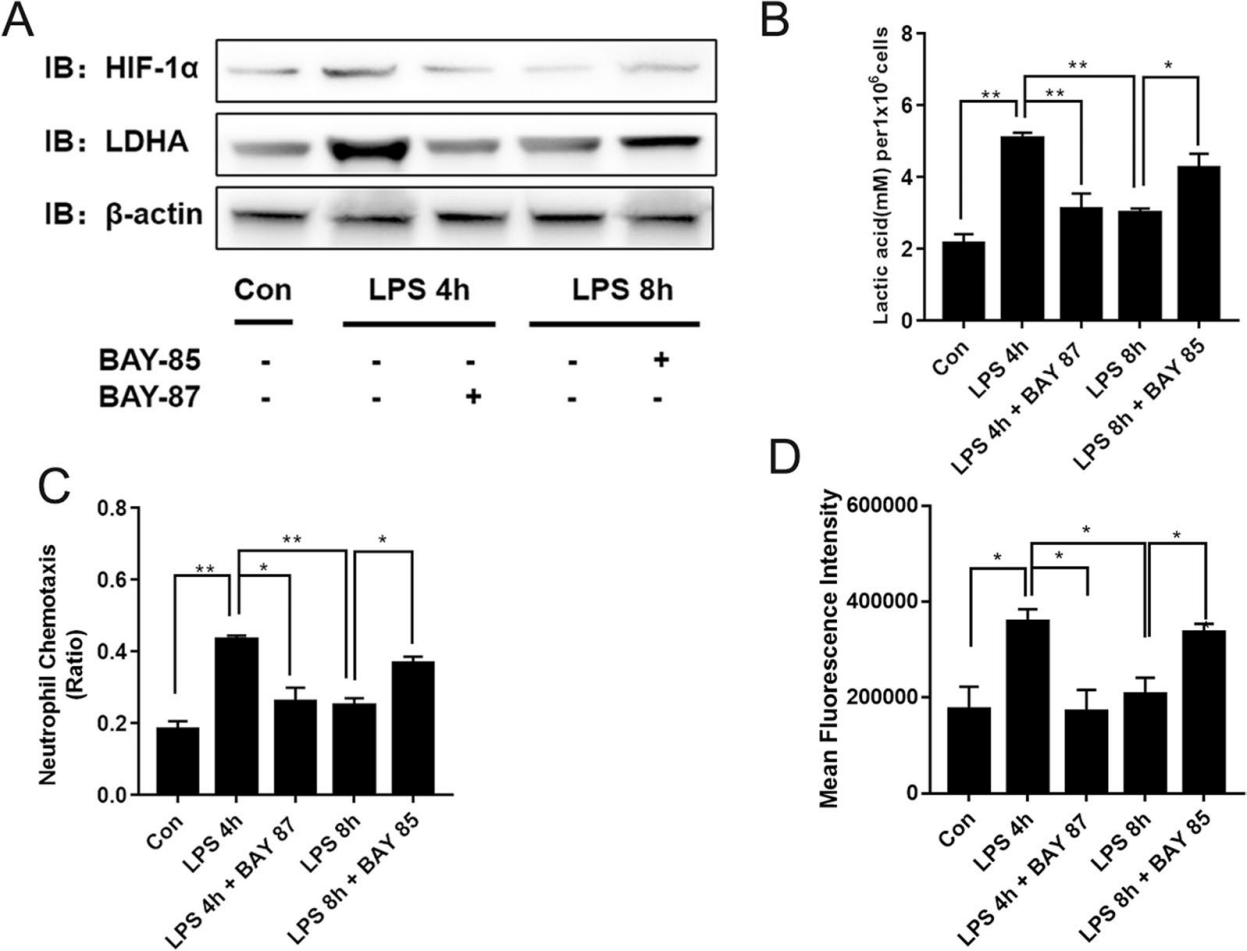
HIF-1α regulates LDHA expression in PMNs during sepsis. The HIF-1α stabilizer BAY-85 and the HIF-1α inhibitor BAY-87 were used to clarify the roles of HIF-1α in the regulation of PMN glycolysis during sepsis. PMNs were stimulated with LPS for 4 h or 8 h in the presence of BAY-85 or BAY-87. Cell lysates were prepared at the indicated time points. **a** Western blot for ΗΙF-1α, LDHA, and actin. **b** *L*-lactate concentrations in PMNs. **c** PMN chemotaxis. **d** PMN phagocytosis. Images are representative of three independent experiments. One-way ANOVA with Brown-Forsythe and Welch ANOVA tests were performed. Error bars represent SD. Data are means ± SD of at least three independent experiments. *P < 0.05, **P < 0.01

HIF-1α plays a central role in the development of myeloid cell-mediated inflammation. The HIF-1α stabilizer BAY-85 and inhibitor BAY-87 were used to explore the role of HIF-1α in PMN glycolysis during sepsis. BAY-85 inhibits HIF proline hydroxylase (HIF-PH) and reduces the degradation of HIF, thereby stabilizing HIF-1α [31, 32]. BAY-87 is a potent and selective HIF-1α inhibitor which was found to inhibit the expression of HIF target genes and the accumulation of HIF-1α protein [33]. The results showed that the levels of LDHA, lactate, chemotaxis, and phagocytosis functions were significantly enhanced in the LPS-tolerant neutrophils upon BAY-85 pretreatment (Fig. 7a–d). In contrast, the levels of glycolysis, chemotaxis, and phagocytosis functions were inhibited in the LPS-activated neutrophils upon BAY-87 pretreatment (Fig. 7b–d). In addition, by knocking down HIF-1α in HL60 cell models, we found that the levels of glycolysis and phagocytosis of neutrophils after LPS stimulation for 2 h were significantly lower (Additional file 9: Fig. S5).

## Discussion

Effective removal of pathogenic bacteria is key to improving the prognosis of sepsis. Neutrophils play vital roles in the clearance of pathogenic bacteria [4]. Neutrophils are required to meet their energy demands at inflamed sites where nutrients may be limited [34]. However, the metabolic characteristics and immunomodulatory pathways of neutrophils during sepsis have not been explored. Our study demonstrated that the inhibition of glycolysis contributed to neutrophil immunosuppression during sepsis and might be controlled by PI3K/Akt-HIF-1α pathway-mediated LDHA downregulation. Walmsley et al. [35] have provided evidence of a specialized metabolism that enables neutrophils to utilize glycogen cycling for energy production, which is essential for neutrophil function and survival, and dysregulation of this metabolism was associated with chronic disease states. Our study revealed more insights into the metabolic characteristics of neutrophils during acute inflammation and focused on glycolysis.

Neutrophils play a crucial role in controlling infection under normal conditions. However, their antimicrobial activity is impaired and their immune responses are dysregulated during sepsis. Neutrophils participate in chemotaxis, phagocytosis, oxidative burst, and neutrophil extracellular traps (NET). These mechanisms help eliminate pathogenic microorganisms [36]. These functions often require cytoskeleton reorganization and metabolic energy. Nevertheless, neutrophils with long lifespans depend mainly on glycolysis. Neutrophils have few functional mitochondria. Hence, their Krebs cycle and oxidative phosphorylation rates are low [20, 37, 38]. Metabolic shifts might introduce heterogeneity in the neutrophil population [39]. However, the relationship between neutrophil metabolism and plasticity merits further investigation.

Technological advancements in metabolomics will help clarify neutrophil immune functions and metabolism. Dysregulation of neutrophil metabolism has been observed in various inflammatory diseases such as diabetes [40, 41], cystic fibrosis [42], lupus [43], and atherosclerosis [44]. However, the association between the metabolic characteristics and the immunomodulatory pathways of neutrophils during sepsis has not been analyzed. In the present study, we used metabolomics to examine the metabolic properties of neutrophils during sepsis. The Warburg Effect, Mitochondrial Electron Transport Chain, Ammonia Recycling, Glycerolipid Metabolism, and De Novo Triacylglycerol Biosynthesis pathways were the most significantly altered during sepsis. We found that lactic acid levels were significantly higher in the neutrophils of infected patients without sepsis than in those of healthy controls. Furthermore, lactic acid levels were significantly lower in the neutrophils of infected patients with sepsis than in those of infected patients without sepsis. For decades, lactate has been considered a waste product of cellular metabolism. However, recent studies have reported lactate to be an essential and novel molecule that affects human immune cell metabolism and may therefore serve as a negative feedback signal limiting glycolysis [45, 46]. Exposure to lactate immediately reduced glycolytic rates and increased oxidative rates in human monocytes in vitro and thereby influenced cellular function [45]. Although metabolism-modulating effects of lactate have been reported, evidence from human neutrophils is scarce. Further studies are required to investigate whether lactate exerts a feedback effect limiting glycolysis in neutrophils.

In vitro, we found that PMNs switched towards aerobic glycolysis when they were activated. Moreover, glycolysis was inhibited in LPS-tolerant neutrophils. In contrast, neutrophilic chemotaxis and phagocytosis functions were inhibited when the glycolysis inhibitor 2-DG abrogated ECAR and lactate production. Thus, we concluded that glycolysis regulated neutrophil immune function in the LPS-tolerant model. Recent studies showed lactate could play an immunosuppressive role in sepsis [47, 48]. Xu et al. reported that lactate might induce immunosuppression by promoting lymphocytic apoptosis in acute septic kidney injury [49]. Colegio et al. reported that lactate could be the primary mediator responsible for promoting M2 macrophage polarization [50]. Lactate treatment also upregulated M2-associated genes and markers by a mechanism dependent on HIF-1α [51]. Recent studies have suggested that lactate may serve as a feedback signal that limits excessive inflammatory response by interfering with cellular metabolism [52, 53]. Selleri et al. reported that lactate induced dose-dependent preferential monocyte differentiation into M2 macrophages by metabolic reprogramming [54]. Besides the anti-inflammatory effects of lactate in murine macrophages, it has been reported that elevated levels of lactate can decrease pro-inflammatory cytokines production in human PBMCs in vitro [53]. However, it is unknown how lactate triggers immunosuppression in neutrophils, which deserves to be explored further.

The changes that occur in metabolism-related enzymes in immunocytes during sepsis play critical roles in the immune function of these cells [55–57]. Neutrophils are highly dependent on glycolysis for energy. Hence, their immune functions such as chemotaxis, phagocytosis, and NET formation are fueled mainly by glycolysis [20, 37, 38]. Here, we observed significant in vitro decreases in lactate and LDHA in LPS-tolerant neutrophils. The results of our metabonomics and transcriptomics analyses were consistent with this phenomenon. Moreover, changes in the expression levels of pyruvate dehydrogenase kinase 1(PDK1), glucose transporter 1(GLUT1), LDHA, and pyruvate kinase M2 (PKM2) regulate macrophage glycolysis, the release of proinflammatory cytokines, and macrophage activation during sepsis [58].

Our subsequent in vitro experiments revealed that pretreatment with BAY-85 to stabilize HIF-1α significantly increased LDHA expression and lactate levels, which further enhanced the chemotactic and phagocytosis functions. PI3K/Akt-HIF-1α pathway was demonstrated to be involved in the expression of LDHA and affect the immune function of neutrophils. The PI3K/Akt pathway plays vital roles in the normal immune function of neutrophils [59, 60]. Oxidative burst and phagocytic activity were significantly reduced in the neutrophils of mice with sepsis when the PI3K pathway was inhibited [61]. These findings were consistent with our results. Other studies showed that HIF-1α plays a crucial role in the development of myeloid cell-mediated inflammation during LPS-induced sepsis [62, 63]. Oncological research has reported that HIF-1α upregulation significantly promoted LDHA expression in bladder cancer cells [64]. HIF-1α upregulation in breast cancer cells inhibited the Warburg effect, enhanced mitochondrial oxidative phosphorylation, induced the accumulation of reactive oxygen species (ROS), and contributed to tumor cell apoptosis [65]. HIF-1α also directly regulated LDHA expression in neuroinflammation [66]. In the microglia of ischemic rat brain tissue, HIF-1α was upregulated, which in turn promoted LDHA expression and aggravated inflammation [66]. Our present study indicated for the first time that HIF-1α may regulate neutrophil functions in sepsis via LDHA. HIF-1α might signify an important and novel therapeutic target to improve neutrophil function during sepsis. Future studies are required to investigate the role of HIF-1α in neutrophils’ immune function in the early stage of sepsis.

## Conclusions

**Fig. 8:**
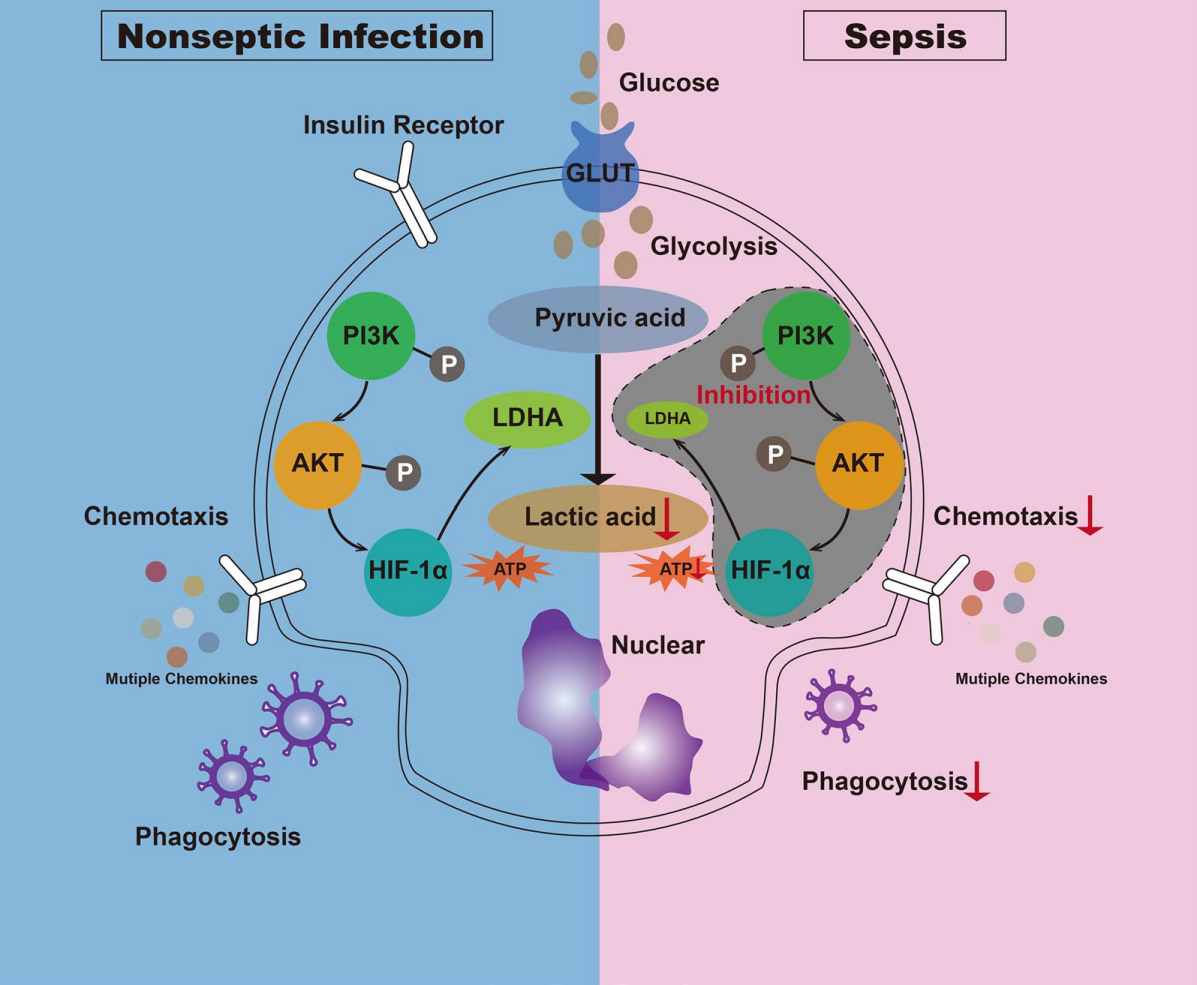
Mechanism of glycolysis-regulated immune effects in neutrophils during sepsis

In conclusion, the inhibition of glycolysis suppressed the immune function of neutrophils during sepsis. This mechanism may have been controlled by PI3K/Akt-HIF-1α pathway-mediated decrease in LDHA expression (Fig. 8). To the best of our knowledge, the present study is the first to explore the mechanism by which glycolysis is inhibited in an in vitro LPS-tolerant model of neutrophils. This discovery could provide a scientific theoretical basis for the management and treatment of patients with sepsis.

## Supplementary Information

**Additional file 1: Table S1.** Reagents and antibodies used in the present study.

**Additional file 2: Table S2.** Characteristics of patients included in the present study.

**Additional file 3: Fig. S1.** Representative diagram of RNA integrity number.

**Additional file 4: Table S3**. Differentially expressed genes (DEGs) between patients with sepsis and healthy controls. This table presents the results of differential expression between patients with sepsis and healthy controls. In total, 677 DEGs with padj < 0.05 were identified. The following information is reported for each gene: Ensembl Gene Name, Fold Change comparing patients with sepsis versus healthy controls, level of statistical significance (padj, qval), genomic coordinates (chromosome_name, start_position, end_position), official gene symbol (external_gene_name), and normalized gene counts for each biological sample.

**Additional file 5: Table S4.** KEGG pathway-enriched clusters obtained from the DEGs list corresponding to patients with sepsis. This table is an extended version of Fig. 3C. All 88 significantly enriched KEGG pathways are described.

**Additional file 6: Fig. S2.** **A** Chemotaxis and **B** phagocytosis in the neutrophils of patients with sepsis, patients with non-septic infection and healthy controls. Data are means ± SD of six independent experiments. Levels of **C** MPO, **D** TNF, **E** IL-1β, **F** IL-6, **G** CX3CR1, **H** CCL2, **I** IL-10 in the LPS-tolerant model. Data are means ± SD of at least three independent experiments. *P < 0.05, **P < 0.01. SS: Sepsis, AA: acute appendicitis.

**Additional file 7: Fig. S3.** L-lactate concentrations in untreated PMNs and in PMNs stimulated with LPS (1 ug/mL) over time. Data are means ± SD of at least three independent experiments. *P < 0.05, **P < 0.01, ***P < 0.001.NS: not significant.

**Additional file 8: Fig. S4.** LDHA regulated phagocytosis and glycolysis in LPS-stimulated neutrophils. **A** Representative flow scatter diagram in control, LPS 4 h, FX-11 + LPS 4 h, and LPS 8 h groups. **B** Representative flow histograms of neutrophils stained with FITC in control, LPS 4 h, FX-11 + LPS 4 h, and LPS 8 h groups. **C** Mean Fluorescence Intensity (MFI) of neutrophils in control, LPS 4 h, FX-11 + LPS 4 h, and LPS 8 h groups. LDHA in the regulation of phagocytosis and glycolysis in LPS-stimulated HL60 cells. HL60 cells were treated with LPS (100 ng/mL) for 2 h or 6 h to build LPS-activated and LPS-tolerant models. After successfully establishing the model, HL60 cells were knocked down of LDHA in the control group and LPS 2 h group. **D** The expression of LDHA in the treatment of LPS, LPS + LDHA siRNA in HL60 cells. **E** Representative flow scatter diagram in control, control + LDHA siRNA, LPS 2 h, LPS 2 h + LDHA siRNA, LPS 6 h, and LPS 6 h + LDHA siRNA groups. **F** Representative flow histograms and **G** MFI of HL60 cells stained with FITC in control, control + LDHA siRNA, LPS 2 h, LPS 2 h + LDHA siRNA, LPS 6 h, and LPS 6 h + LDHA siRNA groups. **H** Lactate concentrations in control, control + LDHA siRNA, LPS 2 h, LPS 2 h + LDHA siRNA, LPS 6 h, and LPS 6 h + LDHA siRNA groups. Data are means ± SD of at least three independent experiments. *P < 0.05, **P < 0.01.

**Additional file 9: Fig. S5.** HIF-1α in the regulation of phagocytosis and glycolysis in LPS-stimulated HL60 cells. **A** Representative flow scatter diagram and **B** Representative flow histograms of HL60 cells stained with FITC in control, control + HIF-1α siRNA, LPS 2 h, LPS 2 h + HIF-1α siRNA, LPS 6 h, and LPS 6 h + HIF-1α siRNA groups. **C** The expression of HIF-1α in the treatment of LPS, LPS + HIF-1α siRNA in HL60 cells. **D** Lactate concentrations in control, control + HIF-1α siRNA, LPS 2 h, LPS 2 h + HIF-1α siRNA, LPS 6 h, and LPS 6 h + HIF-1α siRNA groups. **E** MFI of HL60 cells in control, control + HIF-1α siRNA, LPS 2 h, LPS 2 h + HIF-1α siRNA, LPS 6 h, and LPS 6 h + HIF-1α siRNA groups. Data are means ± SD of at least three independent experiments. *P < 0.05, **P < 0.01, ***P < 0.001.

## Data Availability

The datasets used and/or analyzed in the current study are available from the corresponding authors upon reasonable request.

## Abbreviations

PMNs: Polymorphonuclear neutrophils
LPS: Lipopolysaccharide
OCR: Oxygen consumption rate
ECAR: Extracellular acidification rate
LDHA: Lactate dehydrogenase A
FBS: Fetal bovine serum
LC–MS: Liquid chromatography-mass spectrometry
UPLC-MS: Ultraperformance liquid chromatography coupled to tandem mass spectrometry
PCA: Principal component analysis
OPLS-DA: Orthogonal partial least squares discriminant analysis
VIP: Variable importance in projection
DEMs: Differentially expressed metabolites
BCA: Bicinchoninic acid
BSA: Bovine serum albumin
ECL: Enhanced chemiluminescence
HRP: Horseradish peroxidase
SD: Standard deviation
ANOVA: Analysis of variance
DEGs: Differentially expressed genes
FDR: False discovery rate
PKM: Pyruvate kinase M
HK2: Hexokinase-2
HK3: Hexokinase-3
PDK1: Pyruvate dehydrogenase kinase 1
GLUT1: Glucose transporter 1
