# Supplementary material for: Immune effects of PI3K/Akt/HIF-1α-regulated glycolysis in polymorphonuclear neutrophils during sepsis
Source: Crit Care. 2022 Jan 28;26:29. doi: 10.1186/s13054-022-03893-6 (PMC8796568; doi:10.1186/s13054-022-03893-6)
Supplement: Supplementary file 1 — Additional file 1: Table S1. Reagents and antibodies used in the present study. [file 13054_2022_3893_MOESM1_ESM.docx]

**Supplementary Table S1. Information of reagents and antibodies**

| **Reagent or resource** | **Source** | **Identifier** | |
| --- | --- | --- | --- |
| Antibodies | | | |
| Actin (C-2) | Santa Cruz Biotechnology | Cat#sc-8432;RRID: AB_626630 | |
| Anti-rabbit IgG HRP-linked Antibody | Cell Signaling Technology | Cat#7074S; PRID: AB_2099233 | |
| PKM2 Rabbit Antibody | Cell Signaling Technology | Cat#4053S;RRID: AB_2800137 | |
| LDHA Rabbit Antibody | Cell Signaling Technology | Cat#3582S;RRID: AB_2066887 | |
| HK2 Rabbit Antibody | Cell Signaling Technology | Cat#2867S;RRID: AB_2232946 | |
| HK3 Rabbit Antibody | Abcam | Cat#ab126217;RRID: AB_11127921 | |
| HIF-1α Rabbit Antibody (D1S7W) | Cell Signaling Technology | Cat#36169S;RRID: AB_2799095 | |
| Phospho-PI3 Kinase Class III Antibody (Ser249) | Cell Signaling Technology | Cat#13857S;RRID: AB_2798332 | |
| PI3Kinase p110α Rabbit Antibody (C73F8) | Cell Signaling Technology | Cat#4249S;RRID: AB_2165248 | |
| Phospho-Akt Rabbit Antibody (Ser473) | Cell Signaling Technology | Cat#4060S;RRID:AB_2315049 | |
| Pan-Akt Rabbit Antibody (C67E7) | Cell Signaling Technology | Cat#4691S;RRID: AB_915783 | |
| Chemicals, peptides, and recombinant proteins | | | |
| RPMI1640 | Gibco | | Cat#72400047 |
| [Fetal Bovine Serum](javascript:;) | Gibco | | Cat#10091148 |
| Penicillin-Streptomycin, liquid | Gibco | | Cat#15140122 |
| Phosphate Buffered Saline (PBS) | Gibco | | Cat#10010049 |
| RIPA Lysis and Extraction Buffer | Thermo Fisher Scientific | | Cat#89900 |
| Polymorphprep^TM^ | AXIS-SHIELD | | Cat#1114683 |
| BSA, Fraction V | Fitzgerald | | Cat#30-AB75 |
| LPS from E. coli O8:K27 (S-form) | Adipogen | | Cat#IAX-100-006 |
| DAPI Staining Solution | Abcam | | Cat#ab228549 |
| Insulin | Sigma | | Cat#11061-68-0 |
| 2-DG | Sigma | | Cat#154-17-6 |
| Ly-294002 | MCE | | Cat#HY-10108 |
| fMLP; N-Formyl-MLF | MCE | | Cat#HY-P0224 |
| BAY 87-2243 | Selleck | | Cat#S7309 |
| BAY 85-3934 | Selleck | | Cat#S8138 |
| TRIzol™ Reagent | Thermo Fisher Scientific | | Cat#15596018 |
| DNase/RNase-Free Distilled Water | Thermo Fisher Scientific | | Cat#10977015 |
| XF RPMI Base Medium, PH7.4, 500 ml | Agilent | | Cat#103576-100 |
| XF 1.0M Glucose Solution, 50 ml | Agilent | | Cat#103577-100 |
| XF 100 mM Pyruvate Solution, 50 ml | Agilent | | Cat#103578-100 |
| XF 200 mM Glutamine Solution, 50 ml | Agilent | | Cat#103579-100 |
| Quant-iT PicoGreen dsDNA reagent | Thermo Fisher Scientific | | Cat#P7581 |
| Critical commercial assays | | | |
| Pierce BCA Protein Assay Kit | Thermo Fisher Scientific | | Cat#23252 |
| PVDF Membranes, 0.45 um, 8×10 cm | Merck Millipore | | Cat#IPVH08100 |
| Corning® Cell-Tak™ Cell and Tissue Adhesive, 1mg | Corning | | Cat#354240 |
| Molecular Probes™ FluoSpheres™ Polystyrene Microspheres | Invitrogen | | F13081 |
| XF Cell Mito Stress Test Kit | Agilent | | Cat#103015-100 |
| XF Glycolysis Stress Test Kit | Agilent | | Cat#103020-100 |
| Annexin V-FITC/PI Apoptosis Detection Kit | Solarbio | | Cat#CA1020 |
