# Supplementary material for: Immune effects of PI3K/Akt/HIF-1α-regulated glycolysis in polymorphonuclear neutrophils during sepsis
Source: Crit Care. 2022 Jan 28;26:29. doi: 10.1186/s13054-022-03893-6 (PMC8796568; doi:10.1186/s13054-022-03893-6)
Supplement: Supplementary file 2 — Additional file 2: Table S2. Characteristics of patients included in the present study. [file 13054_2022_3893_MOESM2_ESM.docx]

**Supplemental Table S2. Characteristics of patients included in our study.**

| **Characteristics** | **Healthy Controls** | **Acute Appendicitis** | **Sepsis** |
| --- | --- | --- | --- |
| **Numbers** | 19 | 26 | 14 |
| **Age (year)** | 52±17 | 49±13 | 64±15 |
| **Gender, male/female** | 10/9 | 13/13 | 9/5 |
| **Sites of Infection, n(%)** |  |  |  |
| Lung | - | 0(0) | 2（14.3） |
| Abdominal | - | 26(100) | 12（85.7） |
| Blood | - | 0(0) | 4（28.6） |
| Others | - | 0(0) | 2（14.3） |
| **SOFA Score, median (IQR)** | - | 0(0,1) | 7 (2,11) |
| **Laboratory Tests** |  |  |  |
| CRP (mg/L), mean ± SEM | - | 54 ± 8.9 | 336±181.4 |
| PCT (ng/mL), median (IQR) | - | 3.65 (2.94, 6.45) | 124.2 (0.93, 38.7) |
| WBC (10^9^/L), mean ± SEM | - | 11.4 ± 0.7 | 12.3±7.8 |
| PLT (10^9^/L), mean ± SEM | - | 205.7 ± 11.0 | 164.0 ± 10.2 |
| Lactate (mg/L), mean ± SEM | - | - | 2.8±2.3 |
| **[Immunologic](javascript:;) parameter** |  |  |  |
| Lymphocytes, ×10^9^/L | - | 1.21 ± 0.1 | 0.9 ± 0.1 |
| IgG, mg/dl |  | - | 1049.5±363.5 |
| IgM, mg/dl |  | - | 81.2±50.3 |
| CD3^+^ T lymphocyte count |  | - | 689.1±606.3 |
| CD4^+^ T lymphocyte count | - | - | 381.5±312.9 |
| CD8^+^ T lymphocyte count | - | - | 275.7±310.9 |
| **[Complication](javascript:;)s，n（%）** | - |  |  |
| Acute respiratory failure | - | 0(0) | 9（64.3） |
| Acute cardiac dysfunction | - | 0(0) | 6（42.9） |
| [Acute](javascript:;) [kidney](javascript:;) [injury](javascript:;) | - | 0(0) | 7（50） |
| Acute hepatic insufficiency |  | 0(0) | 6（42.9） |
| **28-day mortality, N(%)** | - | 0(0) | 2 (14.3) |
| **Hospital mortality, N(%)** | - | 0(0) | 2 (14.3) |

Neutrophils(1*10^7^/per sample) were collected from patients [diagnose](javascript:;)d as sepsis or acute appendicitis (without organ impairment) and healthy volunteers to perform metabolomics analysis to find differential metabolites. The characteristics of patients included are [present](javascript:;)ed as listed. SOFA, Sequential Organ Failure Assessment; CRP, C-Reactive Protein; PCT, Procalcitonin; WBC, White Blood Cell count; PLT, Platelet count.
