# Supplementary figures and images for: Immune effects of PI3K/Akt/HIF-1α-regulated glycolysis in polymorphonuclear neutrophils during sepsis
Source: Crit Care. 2022 Jan 28;26:29. doi: 10.1186/s13054-022-03893-6 (PMC8796568; doi:10.1186/s13054-022-03893-6)

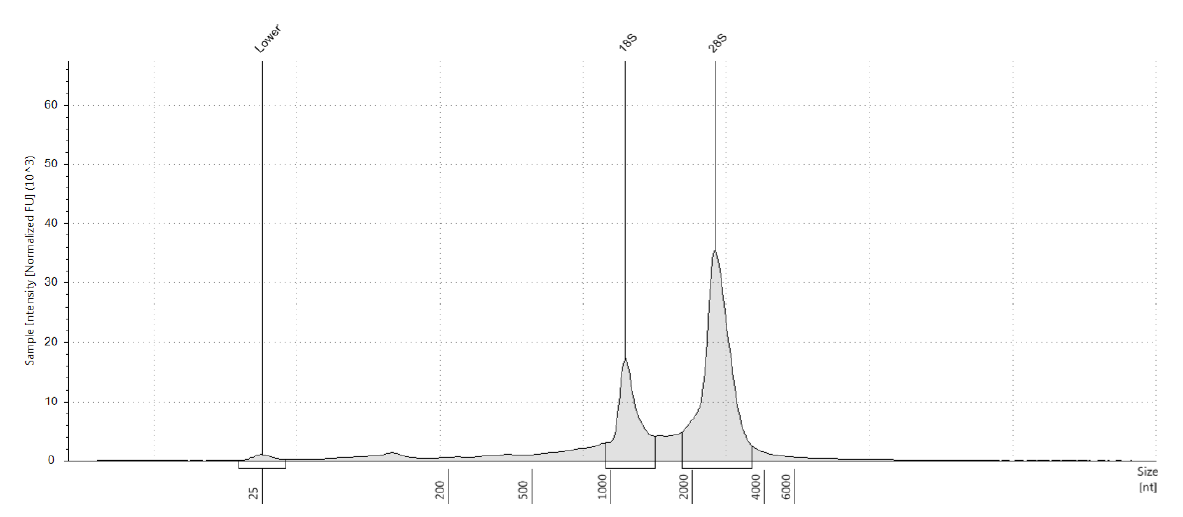

Supplement: Supplementary file 3 — Additional file 3: Fig. S1. Representative diagram of RNA integrity number. [file 13054_2022_3893_MOESM3_ESM.tif]

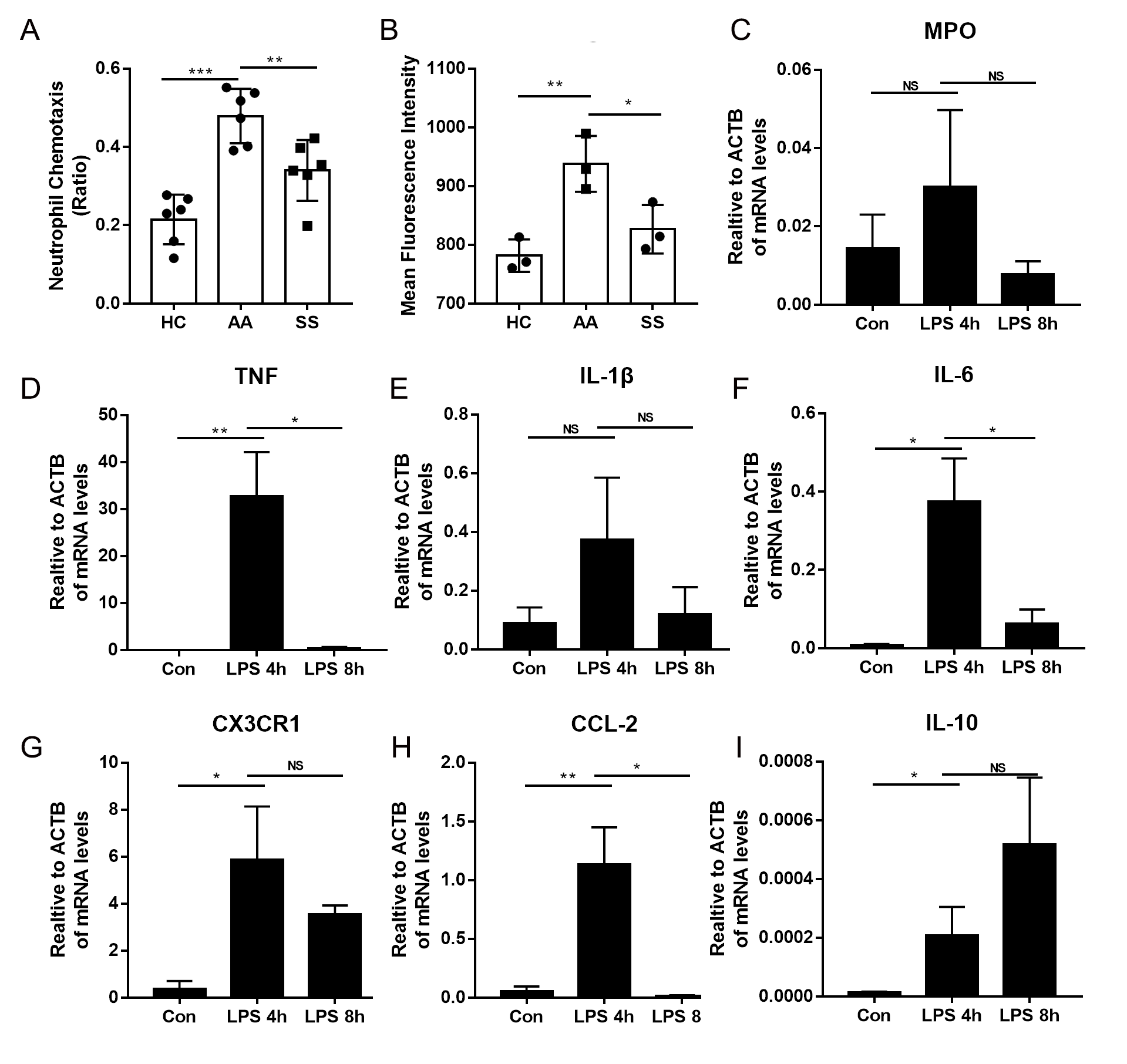

Supplement: Supplementary file 6 — Additional file 6: Fig. S2. A Chemotaxis and B phagocytosis in the neutrophils of patients with sepsis, patients with non-septic infection and healthy controls. Data are means ± SD of six independent experiments. Levels of C MPO, D TNF, E IL-1β, F IL-6, G CX3CR1, H CCL2, I IL-10 in the LPS-tolerant model. Data are means ± SD of at least three independent experiments. *P < 0.05, **P < 0.01. SS: Sepsis, AA: acute appendicitis. [file 13054_2022_3893_MOESM6_ESM.tif]

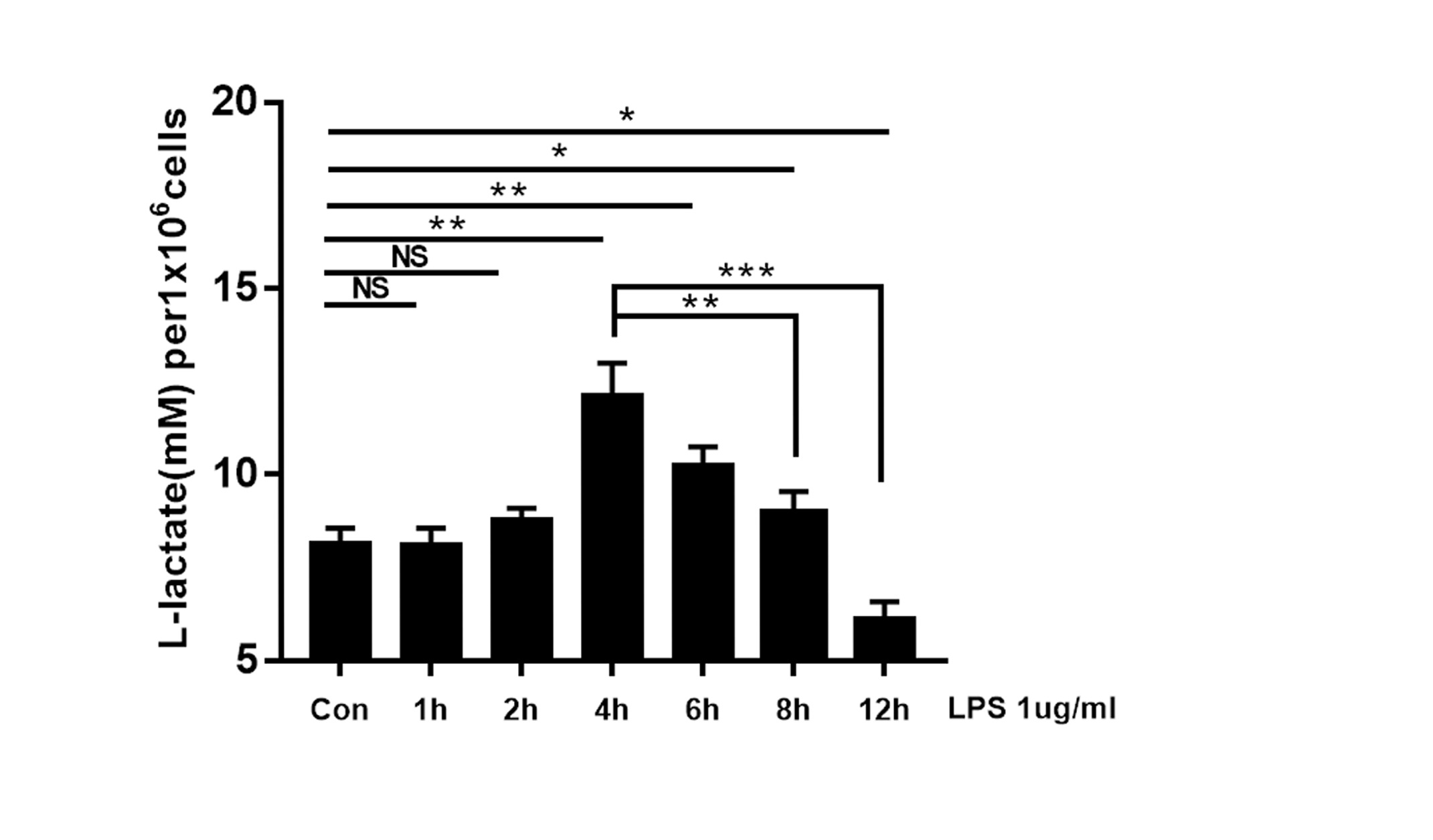

Supplement: Supplementary file 7 — Additional file 7: Fig. S3. L-lactate concentrations in untreated PMNs and in PMNs stimulated with LPS (1 ug/mL) over time. Data are means ± SD of at least three independent experiments. *P < 0.05, **P < 0.01, ***P < 0.001.NS: not significant. [file 13054_2022_3893_MOESM7_ESM.tif]

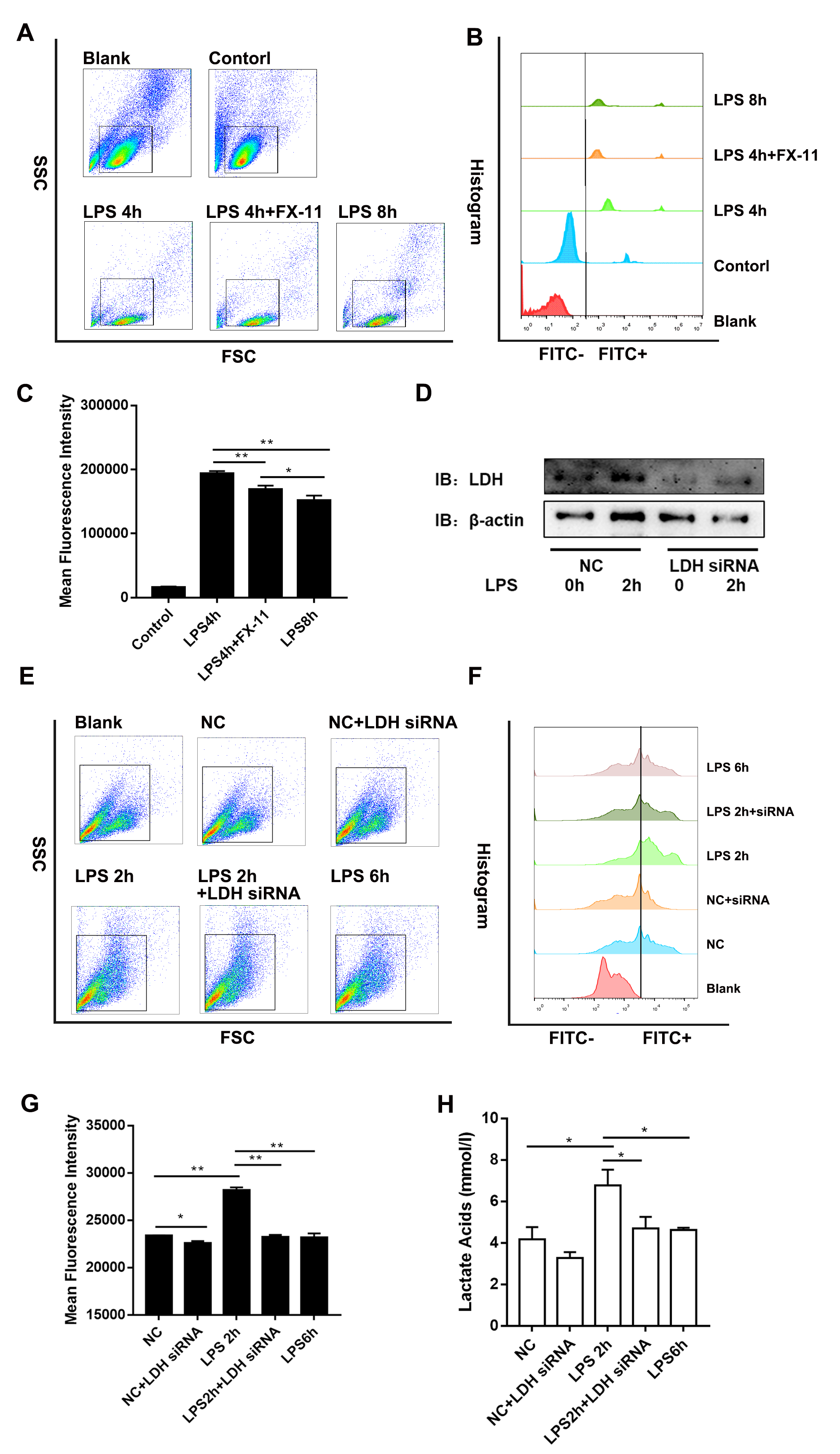

Supplement: Supplementary file 8 — Additional file 8: Fig. S4. LDHA regulated phagocytosis and glycolysis in LPS-stimulated neutrophils. A Representative flow scatter diagram in control, LPS 4 h, FX-11 + LPS 4 h, and LPS 8 h groups. B Representative flow histograms of neutrophils stained with FITC in control, LPS 4 h, FX-11 + LPS 4 h, and LPS 8 h groups. C Mean Fluorescence Intensity (MFI) of neutrophils in control, LPS 4 h, FX-11 + LPS 4 h, and LPS 8 h groups. LDHA in the regulation of phagocytosis and glycolysis in LPS-stimulated HL60 cells. HL60 cells were treated with LPS (100 ng/mL) for 2 h or 6 h to build LPS-activated and LPS-tolerant models. After successfully establishing the model, HL60 cells were knocked down of LDHA in the control group and LPS 2 h group. D The expression of LDHA in the treatment of LPS, LPS + LDHA siRNA in HL60 cells. E Representative flow scatter diagram in control, control + LDHA siRNA, LPS 2 h, LPS 2 h + LDHA siRNA, LPS 6 h, and LPS 6 h + LDHA siRNA groups. F Representative flow histograms and G MFI of HL60 cells stained with FITC in control, control + LDHA siRNA, LPS 2 h, LPS 2 h + LDHA siRNA, LPS 6 h, and LPS 6 h + LDHA siRNA groups. H Lactate concentrations in control, control + LDHA siRNA, LPS 2 h, LPS 2 h + LDHA siRNA, LPS 6 h, and LPS 6 h + LDHA siRNA groups. Data are means ± SD of at least three independent experiments. *P < 0.05, **P < 0.01. [file 13054_2022_3893_MOESM8_ESM.tif]

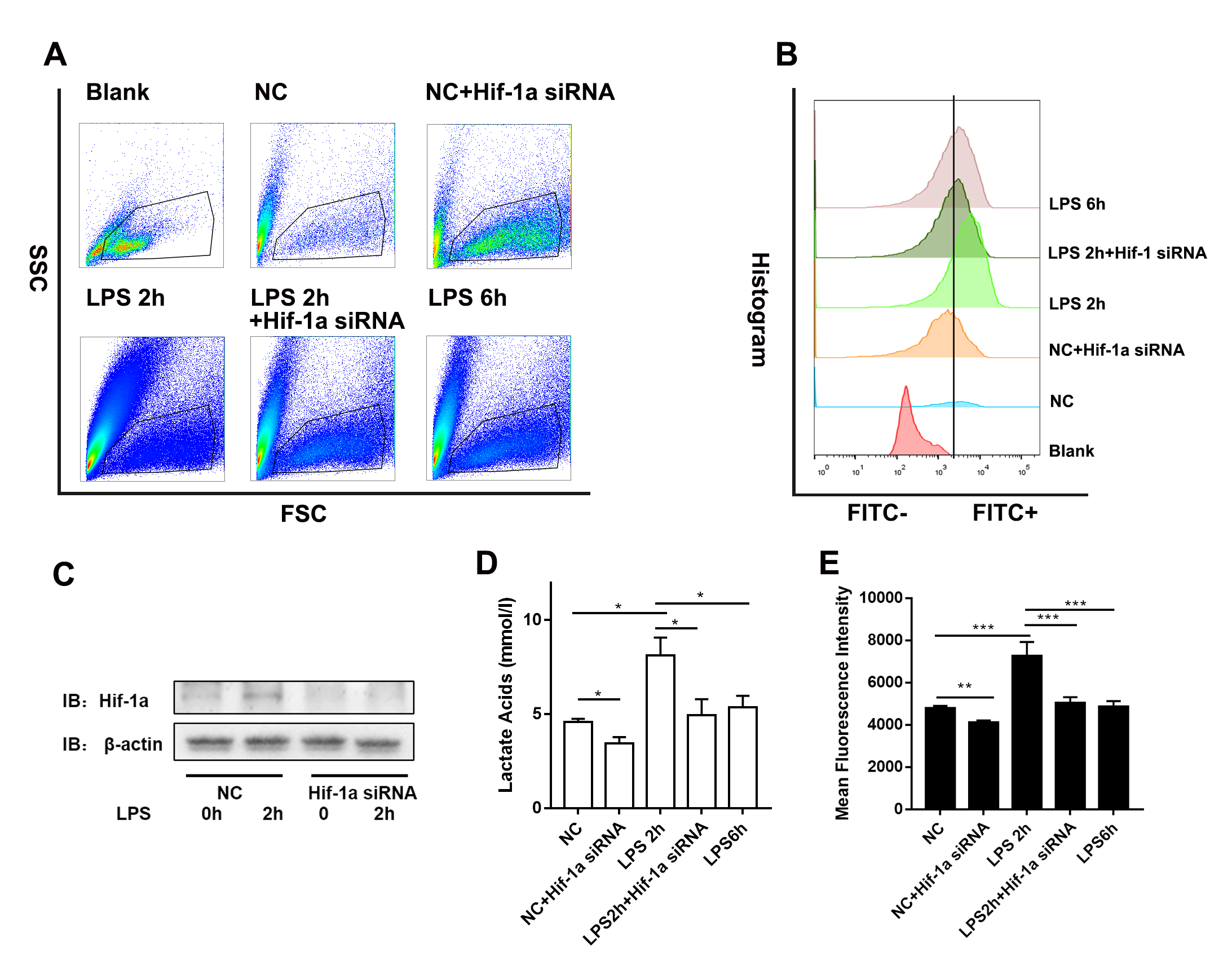

Supplement: Supplementary file 9 — Additional file 9: Fig. S5. HIF-1α in the regulation of phagocytosis and glycolysis in LPS-stimulated HL60 cells. A Representative flow scatter diagram and B Representative flow histograms of HL60 cells stained with FITC in control, control + HIF-1α siRNA, LPS 2 h, LPS 2 h + HIF-1α siRNA, LPS 6 h, and LPS 6 h + HIF-1α siRNA groups. C The expression of HIF-1α in the treatment of LPS, LPS + HIF-1α siRNA in HL60 cells. D Lactate concentrations in control, control + HIF-1α siRNA, LPS 2 h, LPS 2 h + HIF-1α siRNA, LPS 6 h, and LPS 6 h + HIF-1α siRNA groups. E MFI of HL60 cells in control, control + HIF-1α siRNA, LPS 2 h, LPS 2 h + HIF-1α siRNA, LPS 6 h, and LPS 6 h + HIF-1α siRNA groups. Data are means ± SD of at least three independent experiments. *P < 0.05, **P < 0.01, ***P < 0.001. [file 13054_2022_3893_MOESM9_ESM.tif]
